# Antibiotic Resistance Gene Expression in Veterinary Probiotics: Two Sides of the Coin

**DOI:** 10.3390/vetsci12030217

**Published:** 2025-03-02

**Authors:** Ádám Kerek, István Román, Ábel Szabó, Nikolett Palkovicsné Pézsa, Ákos Jerzsele

**Affiliations:** 1 Department of Pharmacology and Toxicology, University of Veterinary Medicine, István utca 2, H-1078 Budapest, Hungary; roman.istvan.laszlo@student.univet.hu (I.R.); szabo.abel@student.univet.hu (Á.S.); palkovicsne.pezsa.nikolett@univet.hu (N.P.P.); jerzsele.akos@univet.hu (Á.J.); 2 National Laboratory of Infectious Animal Diseases, Antimicrobial Resistance, Veterinary Public Health and Food Chain Safety, University of Veterinary Medicine, István utca 2, H-1078 Budapest, Hungary

**Keywords:** veterinary probiotics, benefits, risks, antibiotic resistance genes, ARGs, antimicrobial resistance, AMR, *Bacillus*, *Lactobacillus*, *Enterococcus*, *Pediococcus*

## Abstract

The rapid proliferation of antimicrobial resistance has emerged as one of the most pressing animal and public health challenges of our time. Probiotics, extensively employed in human and veterinary medicine, are instrumental in maintaining a balanced microbiome and mitigating its disruption during antibiotic therapy. While their numerous benefits are well documented, probiotics also present potential risks, notably the capacity to harbor antimicrobial resistance genes. This genetic reservoir could contribute to the emergence and spread of antimicrobial resistance by facilitating the horizontal transfer of resistance genes to pathogenic bacteria within the gut. This review critically examines the presence of antimicrobial resistance genes in commonly used probiotic strains, explores the underlying mechanisms of resistance, and provides a balanced analysis of the benefits and risks associated with their use. By addressing these dual aspects, this paper highlights the need for vigilant evaluation of probiotics to preserve their therapeutic potential while minimizing public health risks.

## Simple Summary

The growing prevalence of antibiotic resistance represents a significant global challenge, demanding urgent and multifaceted solutions. Probiotics, widely recognized for their health benefits in both human and veterinary medicine, remain a topic of debate regarding their efficacy. Emerging evidence indicates that probiotics can harbor antimicrobial resistance genes, raising critical safety concerns. This underscores the necessity for a comprehensive perspective to safeguard the long-term effectiveness of antibiotics. This review consolidates and examines current research on the presence of antimicrobial resistance genes in key probiotic bacterial strains utilized in veterinary medicine.

## 1. Introduction

The widespread use of antibiotics as growth promoters has rapidly driven the emergence of antimicrobial resistance, posing a substantial threat to both human and animal health [1]. To address this escalating concern, the European Union (EU) prohibited the prophylactic use of antibiotics in veterinary practices. Moreover, new EU regulations (2019/6 of the European Parliament and Council of 11 December 2018 on veterinary medicinal products, repealing Directive 2001/82/EC) have further restricted antibiotic usage, particularly in food-producing animals, by limiting prophylactic and metaphylactic applications and banning the use of antibiotics to promote growth. These policy shifts have spurred interest in alternatives which could partially or entirely replace antibiotics. Promising alternatives, which exhibit significant antibacterial efficacy, include antimicrobial peptides [2], parts of plants and plant-derived essential oils [3,4,5], and propolis [6,7,8,9], as well as medium-chain fatty acids and triglycerides [10] or butyrate [11]. However, effective disease control remains paramount to ensuring the responsible use of antibiotics [12] and the choice of therapy based on appropriate pharmacological tests [13]. Probiotics, functioning as alternative growth promoters, have partially supplanted antibiotics by replicating many of their beneficial effects [14]. The growing significance of these alternatives is underscored by research highlighting environmental factors, such as wild birds, in the persistence and spread of antimicrobial resistance [15].

The term “probiotic” was first introduced in 1974, initially describing substances produced by one protozoan that stimulate the growth of another [16]. Today, the World Health Organization (WHO) defines probiotics as “live microorganisms which, when administered in adequate amounts, confer a health benefit on the host” [17]. In veterinary medicine, the most commonly utilized probiotic strains belong to the genera *Lactobacillus*, *Lactococcus*, *Bacillus*, *Enterococcus*, and, in human medicine, *Enterococcus* and *Bifidobacterium* [18,19]. This review focuses on these widely used probiotic strains in the context of their potential role as vectors for antimicrobial resistance genes (ARGs). While not exhaustive, this perspective is essential given the rapid expansion of the probiotic industry into a multi-billion-dollar market, with projected revenues approaching $76 billion by 2026 [20]. Despite their popularity, experiences with probiotics vary, leading to their predominant commercialization as dietary supplements rather than as therapeutic agents [21,22].

According to the recommendations of the WHO, the Food and Agriculture Organization (FAO), and the European Food Safety Authority (EFSA), probiotics must satisfy stringent safety, functional, and technological criteria to ensure their efficacy and reliability [23,24,25]. A summary of these essential requirements is provided in Table 1.

Maintains viability during product processing (e.g., freezing), ensuring delivery of live cultures.

The objective of this study was to conduct a systematic review of the prevalence of ARGs in probiotic strains commonly utilized in veterinary medicine, as documented in the scientific literature. While not exhaustive, we have sought to explore the practical applications of probiotics, highlighting their benefits while addressing potential risks. By adopting a multi-faceted perspective and fostering collaborative critical thinking, this work aims to contribute to the One Health concept, bridging the interconnected domains of animal and public health.

## 2. Benefits and Risks of Probiotics

The efficacy and safety of probiotics remain contentious topics, as research has yet to provide conclusive evidence of their unequivocal benefits or risks. A comprehensive 2011 study found no significant health risks associated with probiotic use in clinical trials, but it also failed to establish their beneficial nature beyond doubt [26]. The reality may lie somewhere between these two extremes.

Probiotics exert positive effects (Table 2) through various mechanisms, including recognition by intestinal epithelial and immune cells via specific receptors [27]. This interaction alters cell signaling pathways and cytokine transcription. Additionally, probiotics influence the immune system through interactions with deoxyribonucleic acid (DNA), cell wall components, and metabolites [28,29]. They produce low-molecular-weight antimicrobial substances, such as bacteriocins (e.g., lactic acid and hydrogen peroxide), which can inhibit pathogen growth [30]. Their beneficial properties have also been documented in vaccination trials, indicating their potential to enhance immune responses and improve vaccine efficacy [31]. Probiotics also contribute to gastrointestinal health by adhering to intestinal epithelial cells, preventing pathogen colonization [32,33]. They can inhibit pathogen invasion [34] and reduce pathogen toxin production [35], and may have anticarcinogenic properties because they neutralize genotoxins [36,37].

If farming, the economic significance of probiotics is particularly pronounced in the poultry industry, which accounts for 45% of probiotic market consumption [40]. As the poultry sector [38] is the second-largest user of antibiotics after the swine industry [25], the potential prophylactic effects of probiotics could significantly decrease the use of antibiotics. Thus, by reducing reliance on antibiotics, probiotics may play a vital role in the sustainability of poultry production. Projections indicate that the global poultry meat import will reach 46.7 million metric tons by 2031 [41], driven by its cost-effectiveness, high protein content, and low-fat benefits. As a result, the use of probiotics in poultry is expected to increase substantially [42]. Experimental studies have demonstrated that pre- and probiotic supplementation in broiler chickens reduces mortality rates [38] and enhances humoral immune responses while improving growth uniformity, compared to controls [31]. Additionally, probiotics have been shown to contribute to a reduction in pollutant gas concentrations by facilitating protein degradation, decomposing animal carcasses and feathers, and minimizing ammonia emissions. These effects not only improve animal welfare, but also enhance overall performance and productivity [39].

Despite their many benefits, probiotics also pose certain risks. They have been implicated in systemic infections in humans, including fungemia caused by *Saccharomyces cerevisiae* and *Saccharomyces boulardii* [43,44,45,46,47], and bacteremia associated with *Lactobacillus acidophilus*, *Lactobacillus casei*, and *Lactobacillus rhamnosus* [48,49,50,51,52]. Sepsis cases linked to *Lactobacillus*, *Bacillus subtilis*, and *Bifidobacterium breve* [53,54,55,56], have also been reported, alongside instances of endocarditis and abscess formation [57,58,59,60]. Adverse metabolic effects represent another area of concern. Clinical studies suggest that probiotics may increase the oxygen demand of intestinal mucosa, potentially leading to ischemia and higher mortality rates in patients with pancreatitis [61]. Overstimulation of innate and adaptive immune responses by probiotics could exacerbate immune activity in certain individuals, potentially triggering autoimmune conditions [62,63].

Furthermore, the gut microbiome serves as a reservoir for ARGs. Research has demonstrated that antibiotic treatment can increase ARG diversity, and subsequent probiotic administration may further expand the resistome. This raises concerns about probiotics impeding the normal recolonization of the gastrointestinal tract following antibiotic therapy [64,65]. *Enterococcus* species, which often carry virulence factors and antimicrobial resistance genes, are particularly controversial as probiotic candidates [66,67,68]. Despite their popularity, only a limited number of studies have provided strong clinical evidence for the health benefits of probiotics, highlighting the need for further investigation into their safety and efficacy [69,70].

## 3. Mechanisms Underlying the Emergence of Antimicrobial Resistance

Mutations serve as a central mechanism in the emergence of antimicrobial resistance (AMR) genes in microorganisms, driving significant genetic variation. These genetic changes directly alter DNA sequences, resulting in new alleles that can be inherited within populations [71]. The environment plays a crucial role in this process, acting as a selective pressure through factors such as antibiotics, temperature, and pH levels, which drive adaptive mutations [72]. A prime example of adaptive mutation occurring in antibiotic-rich environments comes from Zhang et al. (2017), who demonstrated the adaptation of a *Lactobacillus paracasei* strain to amoxicillin and gentamicin, where mutations stabilized once drug resistance was achieved [73]. Similarly, the expression of alkaline shock protein (asp23) has been linked to resistance development [74]. Further studies have identified single-nucleotide polymorphisms (SNPs), insertions, and structural mutations in *Lactobacillus plantarum* that significantly enhanced resistance to gentamicin [75].

Gene flow is another critical pathway for resistance development, involving the exchange of genes or genotypes between species via mechanisms like recombination, mobile genetic elements (MGEs), plasmids, transposons, and genomic islands. Horizontal gene transfer (HGT), a major form of gene flow, acts as a significant evolutionary force in microbial adaptation, though pinpointing specific gene flow events remains challenging [76,77]. Selective pressure facilitates recombination, creating novel genetic combinations that accelerate adaptation. In certain contexts, non-homologous recombination is synonymous with HGT, further spreading resistance genes [78].

The evolution of antibiotic resistance presents profound challenges in microbiology and public health. Microorganisms have developed multiple mechanisms to evade antibiotics, including enzymatic degradation or modification of the antibiotic [79], efflux pump-mediated expulsion [80], mutations altering target sites [81], target replacement [82], protection of the target site [83], reduced membrane permeability to antibiotics, and even complete disappearance of the target molecule [84].

Enzymatic degradation or modification is a common resistance mechanism, often involving structural changes that render drugs ineffective. Examples include covalent bond modifications such as O-phosphorylation, O-ribosylation, O-glycosylation, O-nucleotidylation, and O- or N-acetylation, which inhibit the antibiotic’s ability to bind effectively to its target [85]. Efflux pumps also play a significant role, actively removing antibiotics from bacterial cells. These pumps, frequently encoded on mobile genetic elements [80], show specificity for particular drug groups [86]. Mutations enhancing efflux pump activity or expression can further exacerbate resistance of this type. Alterations in antibiotic target sites are another major resistance mechanism. Mutations can reduce the binding affinity of antibiotics or deactivate regulatory proteins, enhancing resistance. In some cases, bacteria replace antibiotic targets with alternative proteins that maintain functionality but differ structurally [81,87,88], bypassing the antibiotic’s mechanism of action [89,90,91,92,93]. Enzymes like ribosomal methylases protect target sites, preventing effective antibiotic binding [83]. A decrease in antibiotic permeability of cell membranes is another resistance pathway, achieved through changes in the number or affinity of porin channels or via enzymes and proteins that indirectly limit antibiotic entry [84,94]. This mechanism reduces intracellular antibiotic concentrations, diminishing their efficacy.

## 4. Antibiotic Resistance in Probiotics

When assessing the safety of probiotics, particular emphasis should be placed on their potential to transfer ARGs within the gut microbiome. A key prerequisite is that probiotics should not function as reservoirs or vectors for the dissemination of resistance genes [95]. Despite the importance of this concern, global safety standards remain inconsistent and underdeveloped. Nevertheless, various initiatives aim to address this gap. Noteworthy efforts include the ‘Biosafety Assessment of Probiotics used for Human Consumption’ (PROSAFE), the ‘Assessment and Critical Evaluation of Antibiotic Resistance Transferability in Food Chain’ (ACE-ART) project, and the collaboration between the International Organization for Standardization and the International Dairy Federation (ISO–IDF). These programs are focused on reducing the risks associated with the transferability of ARGs via probiotics [95,96]. Subsequent sections of this review will examine the ARGs identified in the most widely utilized probiotic strains. To provide a comprehensive context, it is essential to first explore the broader implications of antimicrobial resistance gene transfer mechanisms, particularly in lactic acid-producing bacteria, which are highly relevant to probiotic applications. This exploration will highlight the critical role of HGT in disseminating ARGs and its associated challenges.

Lactic acid-producing bacteria are particularly associated with the horizontal transfer of ARGs, pathogenicity factors, and metabolic functional genes [78]. A major challenge with HGT lies in the difficulty of accurately identifying and monitoring these genetic exchanges [97]. Among the mechanisms of HGT, plasmid-mediated transfer is predominant, enabling the exchange of resistance genes across diverse taxonomic groups [98]. Insights from functional genomics are poised to become a cornerstone of future food safety measures. Identifying ARGs and ensuring their absence from mobile genetic elements, such as plasmids or bacteriophages, are critical steps. This strategy minimizes the potential for the spread of resistance genes, thereby reducing public health risks [99].

Genome-wide analysis of probiotic bacterial genomes represents an emerging area of research with significant implications. Although still in its early stages, this approach has demonstrated its utility in identifying ARGs within lactate-producing bacteria. For instance, Campedelli et al. (2019) utilized genome-wide analysis to detect resistance genes in lactic acid bacteria, uncovering resistance to several antibiotic classes, including aminoglycosides, tetracyclines, macrolides, lincosamides, and phenicols [100]. Such studies highlight the need for continued advances in genomics to ensure the safety and efficacy of probiotics.

### 4.1. Bacillus amyloliquefaciens

*Bacillus* species, characterized as aerobic, endospore-forming bacteria, have garnered significant attention in recent studies for their promising probiotic properties [101]. Among these, *Bacillus amyloliquefaciens* has demonstrated notable benefits, including enhancing immune responses, improving digestion, and facilitating nutrient absorption through the production of diverse extracellular enzymes [102]. Neverling et al. (2020) demonstrated that *B. amyloliquefaciens* effectively inhibits *Salmonella* colonization in the gastrointestinal tract of broiler chickens [103]. However, this species also harbors ARGs that could pose risks to both animal and public health.

One of the key ARGs identified in *B. amyloliquefaciens* is *clbA*, which encodes a 23S rRNA methyltransferase. This plasmid-encoded gene belongs to the *cfr*-like group and mediates resistance to phenicols, lincosamides, and pleuromutilins via enzymatic inactivation [104]. Additionally, Tojo et al. (2015) reported two strains of *B. amyloliquefaciens* resistant to rifampicin due to the presence of the *rpoB* and *rpsL* genes [105]. The *tetL* gene, responsible for encoding an efflux pump that confers tetracycline resistance, was identified by Nohr-Meldgaard et al. in 2022 [106]. Other ARGs documented in this species include *satA*, which confers enzymatic resistance to aminoglycosides, *lmrB* and *cfrB*, associated with macrolide and lincosamide resistance [107], and *aadK*, which mediates enzymatic inactivation of aminoglycosides [108].

In a recent study employing next-generation sequencing, the *clbA* gene was identified, which plays a significant role in conferring resistance to phenicols, lincosamides, and pleuromutilins. Additionally, the *dfrA43* gene was detected, which provides resistance to diaminopyrimidines [109]. These findings underscore the dual public and animal health significance of these genes, particularly due to the risk of HGT.

### 4.2. Bacillus licheniformis

*Bacillus licheniformis* has gained recognition for its therapeutic potential in addressing gut microbiome dysbiosis [110]. This species is known to harbor the *cat* gene, which encodes an acetyltransferase responsible for resistance to chloramphenicol. Furthermore, it carries *aadK* and *APH* genes, both associated with aminoglycoside resistance [111]. A range of genes linked to macrolide esistance has also been identified in this species, including *ereA*, *ereB*, *ermD*, *ermC*, *ermA*, and *ermB* [111,112,113]. In addition, *bcrA*, *bcrB*, and *bcrC* genes, which encode efflux pumps conferring resistance to bacitracin, have been documented [105,114]. Our findings revealed a total of five ARGs, all located on chromosomal DNA. Among these, we identified previously reported genes such as *aadK*, *bcrA*, *bcrB*, *bcrC,* and *ermD* [109].

### 4.3. Bacillus subtilis

*B. subtilis* is widely recognized for enhancing feed utilization and promoting growth, particularly within the small intestine [115]. Commonly employed in swine farming to encourage growth [116], this bacterium is not traditionally classified as a lactic acid-producing bacteria (LAB). However, it demonstrates a notable capacity for lactic acid production [117]. The species provides numerous benefits, including the synthesis of antimicrobial compounds like bacteriocins and antifungal metabolites [118,119], which effectively inhibit the proliferation of pathogenic bacteria [120]. Moreover, *B. subtilis* can produce quorum-quenching enzymes that degrade autoinducers, thereby suppressing the expression of virulence genes in pathogenic bacteria. Among its antibiotic resistance mechanisms, the *aadK* gene encodes a nucleotidyl transferase that confers resistance to aminoglycosides [121,122]. Additionally, the *APH(5)* phosphotransferase enzyme contributes to aminoglycoside resistance [123]. This species also harbors the *tetK* and *tetL* genes, which encode efflux pumps providing resistance to tetracyclines [124,125,126,127]. The *mprF* gene modifies the membrane’s negatively charged phosphatidylglycerol, leading to resistance against cationic antimicrobial peptides [128,129,130].

Other notable ARGs in *B. subtilis* include the *blt* gene, a multidrug efflux pump linked to fluoroquinolone resistance [131] and the *bmr* gene, another multidrug efflux pump that confers resistance to both fluoroquinolones and chloramphenicol [132,133]. The *lmrB* gene encodes an ATP-binding cassette-type efflux pump, granting resistance to lincosamides [134]. The *mphK* gene encodes a phosphotransferase associated with macrolide resistance [135]. The *vmlR* gene, responsible for ribosomal protection, provides resistance to a broader spectrum of antibiotics, including lincosamides, pleuromutilins, phenicols, tetracyclines, and macrolides [136]. Lastly, the *ykkC* and *ykkD* genes, which encode small multidrug resistance (SMR) type efflux pumps, confer resistance to phenicols, tetracyclines, and aminoglycosides [137].

In a prior study, we analyzed a probiotic preparation containing *B. subtilis* using next-generation sequencing. Our findings revealed a total of eight ARGs, all located on chromosomal DNA. Among these, we identified previously reported genes such as *blt*, *vmr*, *mphK*, *vmlR*, *ykkC*, *ykkD*, *lmrB*, and *mprF* [109].

### 4.4. Enterococcus faecium

*Enterococcus faecium* is extensively used in both veterinary and human healthcare applications [138], although its use remains a subject of debate. Certain strains demonstrate significant probiotic potential, particularly as food additives and therapeutic agents [139]. These strains have been effective in treating diarrheal diseases in young animals and infections caused by feline herpesvirus 11 [140], primarily by functioning as immune system enhancers [141]. However, the application of *E. faecium* is controversial due to its capacity to harbor and disseminate acquired ARGs, raising concerns about the long-term safety of its use [142]. A critical study revealed several ARGs in *E. faecium*, including the *aac(6′)-li* gene, which confers resistance to aminoglycosides; the *msrC* gene, linked to multidrug resistance and ribosomal protection; and the *efmA* gene, which encodes an efflux pump conferring resistance to macrolides and fluoroquinolones [143,144,145,146].

Extensive genome sequencing of *E. faecium* strains isolated from dairy products identified additional ARGs, such as *msrA* and *msrB* (macrolide resistance), *tetK*, *tetL*, and *tetM* (tetracycline resistance), the *cat* gene (chloramphenicol resistance), and the *ermB* gene (macrolide resistance) [147]. Notably, a study investigating a potential probiotic strain of *E. faecium* identified the *vanC1* gene. This gene encodes a D-Ala-D-Ala ligase homologue, enabling the formation of D-Ala-D-Ser, which significantly reduces vancomycin’s efficacy [148].

In a recent study, we employed next-generation sequencing to analyze probiotic formulations containing *E. faecium* approved for use in poultry, specifically focusing on the presence of ARGs. This analysis revealed several ARGs, including *AAC(6′)-Ii* (responsible for enzymatic inactivation of aminoglycosides), *msrC* and *eatAV* (associated with mutations at the target sites of macrolides, lincosamides, pleuromutilins, phenicols, and tetracyclines), as well as *efmA* (encoding an efflux pump for macrolides and fluoroquinolones) [109].

When examining formulations designed for companion animals, in scenarios using the same assay, additional ARGs were identified. These included *AAC(6′)-Ii*, *ermB* (linked to macrolide resistance), and *tetC* and *tetM* (conferring tetracycline resistance) located on plasmids. Notably, the *efmA* gene was detected as a mobile genetic element, further emphasizing the potential for HGT and its implications for resistance dissemination [149].

### 4.5. Lactobacillus acidophilus

*L. acidophilus* is widely recognized both as a major dietary supplement and a natural component of the human microbiota [150,151]. Studies have revealed several ARGs within this species. The *gyrA* gene, for instance, confers resistance to fluoroquinolones by altering the binding site on the alpha subunit of DNA gyrase, an enzyme essential for DNA unwinding [152]. Additionally, the *ermB* gene, responsible for macrolide and lincosamide resistance, operates via a methyltransferase mechanism, rendering these antibiotics ineffective. This was first highlighted by Drago et al. in 2011 [153] and later linked to clindamycin resistance by Aristimuño et al. in 2018 [154]. Furthermore, the *tetM* gene, which plays a pivotal role in conferring resistance to tetracyclines, has also been documented within this species, as substantiated by Cataloluk et al. in 2004 [155]. The ARGs identified in *L. acidophilus* underscore the importance of vigilance when utilizing this probiotic strain to mitigate potential risks associated with antimicrobial resistance, because of the practical importance of the resistance genes described.

### 4.6. Lactobacillus brevis

*Lactobacillus brevis*, commonly found in fermented plants, various foods, and the intestinal tract, exhibits significant resilience to certain antibiotics due to its repertoire of ARGs [156]. Among these, the *ermC* gene plays a critical role, encoding a methyltransferase that confers resistance to macrolides and lincosamides by modifying their target sites [157]. Additionally, *L. brevis* harbors the *gyrA* gene, which facilitates resistance to fluoroquinolones by altering the binding site of DNA gyrase, the drug’s target enzyme [158]. Another important ARG is the *parC* gene, which encodes a subunit of topoisomerase IV. Mutations in *parC* disrupt fluoroquinolones’ ability to inhibit DNA synthesis, further enhancing the species’ resistance profile [158,159]. The ARGs identified in *L. brevis* are providing a comprehensive overview of this species’ resistance mechanisms and their implications for both food safety and probiotic applications.

### 4.7. Lactobacillus buchneri

A focused investigation into *Lactobacillus buchneri* revealed the absence of tetracycline resistance genes within the studied strains, emphasizing the variability in antibiotic resistance profiles among probiotic species [160]. This discovery highlights the necessity for species-specific research to comprehensively evaluate the safety and efficacy of probiotics. Understanding such distinctions is crucial for assessing potential health implications, especially in the context of antibiotic resistance dissemination. These findings contribute to the growing body of evidence that supports tailored approaches in the development and application of probiotic therapies.

### 4.8. Lactobacillus fermentum

*Lactobacillus fermentum*, a vital member of the gut microbiome, is commonly found in both the intestinal tract and the oral cavity, where it contributes to dental health by preventing tooth decay [161]. Research has identified several ARGs in this species. The *ermB* gene, which encodes a methyltransferase, renders macrolide and lincosamide antibiotics ineffective. Additionally, the *ANT(6)* and *aadA* genes have been reported to inactivate aminoglycosides through nucleotidylation [154]. Macrolide resistance is further supported by the *msrC* gene, which provides ribosomal protection. Tetracycline resistance in *L. fermentum* is primarily mediated by the *tetK* and *tetL* genes, both of which encode efflux pumps [162]. Subsequent studies have confirmed the presence of the *tetK* gene, reinforcing its significance in tetracycline resistance [163].

### 4.9. Lactobacillus plantarum

*Lactobacillus plantarum* is widely distributed in fermented foods, the intestinal tract, and various probiotic formulations [164]. Its applications extend beyond human nutrition, playing a pivotal role in animal feed as a safe and cost-effective method to reduce economic losses associated with mycotoxin contamination [165]. In the poultry industry, *L. plantarum* demonstrates potential for mitigating ammonia emissions, thereby enhancing environmental sustainability [166]. Additionally, this species has been shown to elevate the expression of tight junction proteins, strengthening the intestinal barrier function [167]. It is also implicated in the upregulation of host defense peptides, thereby fortifying the immune system [168].

*L. plantarum* harbors numerous ARGs that confer resistance to various antibiotic classes. The *ermB* gene is associated with macrolide and lincosamide resistance through a methyltransferase mechanism [153,162]. Fluoroquinolone resistance is linked to the *gyrA* gene, while the *aadE* (ANT(6)) gene inactivates aminoglycosides via nucleotidylation [152]. Similarly, the *aadA* gene also confers aminoglycoside resistance [169]. Resistance to chloramphenicol is mediated by the *cat* gene, which encodes an acetyltransferase, while the *tetS* gene prevents tetracycline antibiotics from impairing ribosome function [154]. Beta-lactam resistance is facilitated by the *blaZ* gene, which encodes a class A beta-lactamase enzyme [157], while the *tetK* and *tetL* genes mediate tetracycline efflux [162]. Ribosomal protection, contributing to additional tetracycline resistance, is provided by the *tetM* and *tetW* genes [163]. Lastly, the *aac(6′)-aph(2′)* gene, which produces an enzyme that inactivates aminoglycosides through acetylation, has been identified [169,170].

### 4.10. Lactobacillus rhamnosus

*L. rhamnosus* is widely recognized for its therapeutic potential, particularly in addressing gastrointestinal disorders. Its ability to inhibit pathogenic microorganisms in vitro and its immunostimulatory properties further underscore its value as a probiotic [171]. Comparative studies between resistant and more susceptible strains have identified the presence of the *emrA*, *emrB*, and *emrC* genes. These genes encode methyltransferase enzymes that confer resistance to macrolide antibiotics by modifying the target site, thereby preventing effective antibiotic binding [172].

### 4.11. Pediococcus acidilactici

*Pediococcus acidilactici* is renowned for its ability to produce antimicrobial compounds, making it a valuable addition to probiotic formulations [173]. Members of this genus have demonstrated a unique capability to suppress the expression of the *stx2a* gene responsible for the production of Shiga toxin 2 (STX2A) in *Escherichia coli*, thereby potentially mitigating its virulence [174]. Additionally, the *ermB* gene, which confers resistance to macrolides through a methyltransferase mechanism, has been identified in studies exploring antimicrobial resistance gene expression in lactic acid bacteria associated with wine production [170].

### 4.12. Pediococcus pentosaceus

*Pediococcus pentosaceus* has gained prominence as a versatile probiotic with applications spanning food, plant, and animal products [175]. However, within this species, the *ermB* gene, which confers macrolide resistance via a methyltransferase mechanism, and the *msrC* gene, also linked to macrolide resistance, have been identified. Furthermore, the presence of the *tetK* and *tetL* genes, encoding efflux pumps that mediate tetracycline resistance, has been documented [162]. Another study revealed the *aac(6′)-aph(2′)* gene, responsible for aminoglycoside resistance through acetylation [170].

In the context of artisan cheese production, research identified the *vanA* and *vanC1* genes, which confer glycopeptide resistance. The *tetO* gene (tetracycline resistance), the *vatE* gene (resistance to macrolides and lincosamides), and the *bcrB* gene (bacitracin resistance) were also reported. The *vanA* gene encodes a D-Ala-D-Ala ligase homologue that synthesizes D-Ala-D-Lac, thereby reducing vancomycin binding by facilitating an alternative pathway for peptidoglycan synthesis [176,177]. Similarly, the *vanC1* gene is involved in the synthesis of D-Ala-D-Ser [178,179]. The *tetO* gene encodes a ribosomal protective protein against tetracycline [180,181], while the *vatE* gene, a transposon-mediated acetyltransferase, contributes to streptogramin resistance [182]. The *bcrB* gene, encoding an efflux pump, provides bacitracin resistance [114].

The comprehensive details of ARGs identified in the probiotics examined in this study are summarized in Table 3.

In lactic acid bacteria, genomic evolution has been significantly shaped by genetic events such as genome reduction, HGT, and gene duplication. These adaptations have facilitated their transition to nutrient-rich environments through both the loss of non-essential genes and the acquisition of advantageous ones [183]. This process, integral to niche adaptation, allows lactic acid bacteria to thrive in new environments via HGT mechanisms enabled by bacteriophages, transposons, and other mobile genetic elements [184].

Lactic acid-producing bacteria have been utilized for thousands of years in the production of fermented foods [185]. These organisms have long served as starter cultures and probiotics, offering significant benefits to consumers [17]. With the advent of genomics, an increasing wealth of genomic data has enhanced our understanding of their biology and functionality [186].

Adaptive laboratory evolution has emerged as a powerful and widely used tool for unraveling the mechanisms of genome evolution and adaptation, enabling the study of functional changes under specific environmental conditions [187]. Numerous studies have highlighted the role of antibiotic-containing environments as a key selective pressure, driving the evolution of antibiotic resistance [73,74,75]. Such selection pressures are critical evolutionary drivers, shaping the adaptability of lactic acid-producing bacteria.

Genetic analyses indicate that some species, such as *L. acidophilus* and *L. fermentum*, possess open pan-genomes, which continue to expand as new genomes are sequenced. This is likely a result of HGT, facilitating the acquisition of novel genes and perpetuating a continuous evolutionary process [188,189]. Conversely, other species, such as *L. plantarum*, exhibit closed pan-genomes, reflecting limited genetic diversity and HGT within the species [190]. Moving forward, the creation of comprehensive, publicly accessible genome databases for these organisms is paramount. Such resources would enable researchers to assemble high-quality genomes and develop sophisticated bioinformatics tools, paving the way for cutting-edge studies in microbial genomics [191]. These efforts will not only deepen our understanding of lactic acid bacteria but also enhance their application in food production, health promotion, and beyond.

## 5. Conclusions

While the occurrence of ARGs in the probiotic strains examined in this study appears limited, their implications are significant, particularly from a One Health perspective. Probiotics, as living microbial supplements, play a vital role in gut health, yet their potential to harbor and transfer ARGs within the gut microbiome remains a concern [192].

For food-producing animals, stringent antimicrobial susceptibility testing is essential to ensure the safety of probiotic strains [193], as mobile genetic elements may facilitate the horizontal transfer of resistance genes. Notably, *L. acidophilus* has been reported to acquire vancomycin resistance genes from *Enterococcus* species [194], and a link has been established between E. faecium probiotics and vancomycin-resistant Enterococcus (VRE) strains associated with nosocomial infections [195].

Despite their widespread use as antibiotic alternatives in livestock production [196], probiotics present a paradoxical risk, potentially contributing to AMR dissemination. While certain strains, such as *B. licheniformis*, may help reduce gut ARG diversity [192,197], others, like *Bacillus coagulans*, have been associated with an increased prevalence of aminoglycoside resistance genes [198]. The robust sporulation capabilities of *Bacillus* species, while beneficial for survival, may also amplify ARG dissemination risks [199]. Conversely, *Pediococcus* species, with their inherent tolerance to osmotic, pH, and temperature variations, appear to carry fewer known resistance genes, making them a potentially safer choice for probiotic development [200,201].

Moving forward, interdisciplinary research should prioritize large-scale genomic and phenotypic studies to assess the mobility of ARGs and the risk of horizontal gene transfer (HGT). Integrating whole-genome sequencing, minimum inhibitory concentration (MIC) assays, and gene expression profiling will be essential in evaluating the safety and efficacy of probiotics. A multidisciplinary, evidence-based approach will reinforce their role as sustainable tools in combating antimicrobial resistance while ensuring public and animal health safety.

## Figures and Tables

**Table 1:** Basic criteria for probiotics. This table summarizes the fundamental technological, functional, and safety standards that probiotic strains are required to meet, as outlined in referenced guidelines [23,24,25].

Criteria	
**Safety**	Derived from healthy animals or humans to ensure a safe starting point.
	Possesses well-defined phenotypic and genotypic characteristics for accurate classification.
	Incapable of causing disease in hosts, ensuring safety.
	Does not metabolize bile salts, indicating a benign interaction with the digestive system.
	Encodes resistance genes strictly as non-mobile elements, reducing horizontal gene transfer risk.
**Functionality**	Effectively competes within the gut microbiome, ensuring establishment and persistence.
	Exhibits strong antagonistic effects against pathogens, bolstering host defense mechanisms.
	Survives low gastric pH and bile, ensuring passage through the gastrointestinal tract.
	Successfully colonizes targeted gut areas, achieving desired health benefits.
	Tolerates bactericidal substances and acids produced by the gut microbiome.
**Technology Usability**	Capable of production on a large scale, supporting commercial viability.
	Maintains viability during product processing (e.g., freezing), ensuring delivery of live cultures.
	Exhibits a high survival rate throughout marketing and storage, ensuring product efficacy until consumption.
	Does not adversely affect the organoleptic properties of food products, maintaining consumer acceptance.
	Demonstrates genetic stability and resistance to bacteriophages, ensuring consistent quality and performance.

**Table 2:** Proven health benefits of probiotics.

Effects	Description	References
Modulation of the immune system	Probiotics influence the immune response through interactions with DNA, cell wall components, and metabolites, contributing to immune regulation.	[28,29]
Enhancement of vaccine efficacy	Probiotics have demonstrated immunomodulatory properties in vaccination trials, enhancing immune responses and improving vaccine effectiveness.	[31]
Inhibition of pathogen growth	Probiotics produce low-molecular-weight antimicrobial compounds, such as bacteriocins, lactic acid, and hydrogen peroxide, which can inhibit pathogens.	[30]
Prevention of pathogen adhesion	By adhering to intestinal epithelial cells, probiotics prevent pathogen colonization, thereby promoting gastrointestinal health.	[32,33]
Reduction of pathogen toxin production	Probiotics can inhibit pathogen invasion and reduce pathogen toxin production, mitigating harmful effects.	[34,35]
Anticarcinogenic properties	Probiotics may neutralize genotoxins, thereby exerting potential anticarcinogenic effects.	[36,37]
Improvement in growth performance	Probiotics enhance humoral immune responses while promoting growth uniformity and weight gain compared to untreated controls.	[31]
Reduction in mortality rates	The supplementation of prebiotics and probiotics in broiler chickens has been associated with lower mortality rates.	[38]
Environmental benefits	Probiotics contribute to reducing pollutant gas concentrations by facilitating protein degradation, decomposing animal carcasses and feathers, and minimizing ammonia emissions.	[39]

**Table 3:** The antimicrobial resistance genes identified in major probiotic strains are presented along with their respective mechanisms of resistance and the antibiotic classes affected upon their expression. This comprehensive analysis highlights the functional pathways through which these genes confer resistance, offering insights into the potential implications for both clinical and veterinary applications.

Mechanisms	Subgroups	Genes	Antibiotic Resistance	Bacteria	References
Antibiotic efflux	class ABC	*lmrB*	lincosamide	*Bacillus amyloliquefaciens*	[104]
			macrolide		
			lincosamide	*Bacillus subtilis*	[109,134]
		*bcrB*	peptide	*Pediococcus pentosaceus*	[176,177]
				*Bacillus licheniformis*	[105,109]
		*bcrA*			
		*bcrC*			
	class MFS	*tetL*	tetracycline	*Bacillus amyloliquefaciens*	[106]
				*Bacillus subtilis*	[124,125]
				*Enterococcus faecium* *Pediococcus pentosaceus* *Lactobacillus plantarum* *Lactobacillus fermentum*	[147]
		*tetK*			[162]
					[162]
					[162,163]
				*Bacillus subtilis*	[124,125]
		*tetC*		*Enterococcus faecium*	[149]
		*bmr*	fluoroquinolone	*Bacillus subtilis*	[109,133]
			phenicol		
		*efmA*	macrolide	*Enterococcus faecium*	[109,146,149]
			fluoroquinolone		
		*blt*		*Bacillus subtilis*	[109,131]
	class SMR	*ykkC*	phenicol		[109,137]
		*ykkD*	tetracycline		
			aminoglycoside		
Antibiotic inactivation	acetyltransferase	*aac(6′)-li*	aminoglycoside	*Enterococcus faecium*	[109,146,149]
		*cat*	phenicol	*Lactobacillus plantarum*	[154]
		*aac(6′)-aph(2′)*	aminoglycoside		[169,170]
				*Pediococcus pentosaceus*	[170]
		*vatE*	streptogramin		[176,177]
	class A beta-lactamases	*blaZ*	penam	*Lactobacillus plantarum*	[157]
	ANT(3″)	*aadA*	aminoglycoside	*Lactobacillus fermentum*	[154]
	nucleotidyltransferase	*ANT(6)*			
		*aadE (ANT(6))*		*Lactobacillus plantarum*	[152]
		*aadA*			[169]
Antibiotic target alternation	acetyltransferase	*satA*	aminoglycoside	*Bacillus amyloliquefaciens*	[107]
		*cat*	phenicol	*Bacillus licheniformis*	[111]
			phenicol	*Enterococcus faecium*	[147]
	beta-subunit of RNA polymerase	*rpoB*	rifamycin	*Bacillus amyloliquefaciens*	[105]
	D-Ala-D-Ser ligase	*vanC1*	glycopeptide	*Enterococcus faecium*	[148]
				*Pediococcus pentosaceus*	[176,177]
	defensin	*mprF*	peptide	*Bacillus subtilis*	[109,130]
	DNA topoisomerase	*gyrA*	fluoroquinolone	*Lactobacillus acidophilus*	[152]
				*Lactobacillus plantarum*	[152]
		*parC*		*Lactobacillus brevis*	[158,159]
	esterase	*ermA*	macrolide	*Bacillus licheniformis*	[112]
		*ereA*			
		*ereB*			
	ligase	*vanA, vanC*	glycopeptide	*Pediococcus pentosaceus*	[176,177]
	methyltransferase	*cfrB*	lincosamide	*Bacillus amyloliquefaciens*	[107]
			macrolide		
		*clbA*	phenicol		[104,109]
			lincosamide		
			pleuromutilin		
		*ermA*	macrolide	*Lactobacillus rhamnosus*	[172]
				*Bacillus licheniformis*	[112]
		*ermD*			[109,112]
		*ermB*			[113]
				*Enterococcus faecium*	[147,149]
				*Lactobacillus acidophilus*	[153]
			lincosamide		
			macrolide	*Lactobacillus fermentum*	[154]
			lincosamide		
			macrolide	*Lactobacillus plantarum*	[153]
			lincosamide		
			macrolide	*Pediococcus acidilactici*	[170]
			lincosamide		
				*Lactobacillus rhamnosus*	[172]
			macrolide	*Pediococcus pentosaceus*	[162]
		*ermC*		*Bacillus licheniformis*	[113]
				*Lactobacillus brevis*	[157]
			lincosamide, macrolide		
				*Lactobacillus rhamnosus*	[172]
	nucleotidyltransferase	*aadK*	aminoglycoside	*Bacillus amyloliquefaciens*	[108]
				*Bacillus subtilis*	[121,122]
				*Bacillus licheniformis*	[109,112]
	phosphotransferase	*rphB*	rifamycin	*Bacillus amyloliquefaciens*	[107]
		*APH*	aminoglycoside	*Bacillus licheniformis*	[112]
		*APH(5)*		*Bacillus subtilis*	[123]
	phosphotransferase	*mphK*	macrolide		[109,135]
	ribosomal protein	*rpsL*	aminoglycoside	*Bacillus amyloliquefaciens*	[105]
Antibiotic target protection	30S ribosomal subunit	*tetS*	tetracycline	*Lactobacillus plantarum*	[154]
		*tetM*			[163]
		*tetW*			
	ABC-F ATP-binding casette	*vmlR*	lincosamide	*Bacillus subtilis*	[109,136]
			pleuromutilin		
			phenicol		
			tetracycline		
			macrolide		
		*msrC*		*Lactobacillus fermentum*	[162,163]
				*Pediococcus pentosaceus*	[162]
			lincosmaide	*Enterococcus faecium*	[109,144]
			pleuromutilin		
			macrolide		
		*eatAV*	pleuromutilin		[109]
		*msrA*	phenicol		[147]
		*msrB*	tetracycline		
	ribosoma protection protein	*tetO*		*Pediococcus pentosaceus*	[176,177]
		*tetM*		*Lactobacillus acidophilus*	[155]
				*Enterococcus faecium*	[147,149]
Antibiotic target replacement	dihydrofolate reductase	*dfrA43*	diaminopyrimidine	*Bacillus amyloliquefaciens*	[109]
				*Bacillus licheniformis*	[109]
Reduced permeability	resistance protein	*tmrB*	nucleosides		[109]

ABC—ATP-binding cassette; MFS—major facilitator superfamily; SMR—small multidrug resistance.

## Data Availability

Not applicable.

## Abbreviations

The following abbreviations are used in this manuscript:

AMR	Antimicrobial resistance
ARGs	Antimicrobial resistance genes
*B. amyloliquefaciens*	*Bacillus amyloliquefaciens*
*B. licheniformis*	*Bacillus licheniformis*
*B. subtilis*	*Bacillus subtilis*
DNA	Deoxyribonucleic acid
*E. faecium*	*Enterococcus faecium*
EU	European Union
HGT	Horizontal gene transfer
*L. acidophilus*	*Lactobacillus acidophilus*
*L. brevis*	*Lactobacillus brevis*
*L. fermentum*	*Lactobacillus fermentum*
*L. plantarum*	*Lactobacillus plantarum*
*L. rhamnosus*	*Lactobacillus rhamnosus*
*P. acidilactici*	*Pediococcus acidilactici*
*P. pentosaceus*	*Pediococcus pentosaceus*
WHO	World Health Organization
